# Microbiota in Goat Buck Ejaculates Differs Between Breeding and Non-breeding Seasons

**DOI:** 10.3389/fvets.2022.867671

**Published:** 2022-05-13

**Authors:** María Lorena Mocé, Inés Carolina Esteve, Sara Pérez-Fuentes, Ernesto A. Gómez, Eva Mocé

**Affiliations:** ^1^Department of Animal Production and Health, Veterinary Public Health and Food Science and Technology (PASAPTA), Facultad de Veterinaria, Universidad Cardenal Herrera-CEU, CEU Universities, Valencia, Spain; ^2^Unidad Asociada UCH-CEU – IVIA, Valencia, Spain; ^3^Centro de Investigación y Tecnología Animal, Instituto Valenciano de Investigaciones Agrarias, Valencia, Spain

**Keywords:** microbiota (16S), microbiome, anestrous, reproductive season, caprine, sperm, semen, ejaculate

## Abstract

Changes in semen microbiota are associated with alterations to sperm quality and fertility. However, the microbiota from most livestock species has not yet been studied. Goats are seasonal breeders, but semen microbiota has never been described in this species, and it is unknown how seasonality affects it. Our study objective is 2-fold: to describe the microbiota in goat buck ejaculates and to determine if it differs between breeding and non-breeding seasons. Semen from six males of the Murciano-Granadina breed was collected during both seasons. Two replicates were performed per male and season on different days. The microbiota was characterized by genomic sequencing technology. Sperm quality was also evaluated. Repetition was not significant for the studied variables. Sperm velocities were higher for the breeding than for the non-breeding season. The ejaculates from both seasons also differed in the proportion of apoptotic spermatozoa. The five dominant phyla were Firmicutes, Proteobacteria, Fusobacteria, Actinobacteria, and Bacteroidetes during the breeding season and Firmicutes, Proteobacteria, Actinobacteria, Bacteroidetes, and Cyanobacteria during the non-breeding season. The dominant genus during both seasons was *Ureaplasma*. Differences in microbial community structure (the beta diversity) were found. A decrease in the relative abundance of the genus *Faecalibacterium* and an increase in the genera *Sphingomonas* and *Halomonas* were observed in the ejaculates collected during the breeding season. *Sphingomonas* and *Faecalibacterium* abundance favorably and unfavorably correlated with sperm quality, respectively. In conclusion, the semen microbiota from goat bucks varies between breeding and non-breeding seasons, and the microbiota remains stable for 7 days within a season. In addition, the genera *Sphingomonas* and *Faecalibacterium* could be possible biomarkers of semen quality in goat bucks. These results contribute to an in-depth understanding of the effects of reproductive seasonality on goat buck ejaculates.

## Introduction

Recent studies that applied advanced sequencing techniques have suggested that, far from semen being sterile, it harbors its microbial community, and changes in semen microbiota are associated with alterations to semen quality and fertility status in men (1–3). They indicate that the dominant phyla of the seminal microbiota are Firmicutes (~50%) and Proteobacteria (~25%), and the remaining ~25% are Actinobacteria and Bacteroidetes (2).

In humans, the differential abundance of specific bacterial genera in semen suggests that the microbiota might impact sperm quality (4–8). *Pseudomonas* and *Prevotella* are predominant in low-quality ejaculates (4, 7). Monteiro et al. (6) observed that an increase in the *Pseudomonas, Neisseria*, and *Klebsiella* pathogens correlates with seminal hyperviscosity and oligoasthenoteratozoospermia. *Lactobacillus*-predominant ejaculates have been associated with healthy semen (4, 7), although there is some controversy between studies because other authors have observed that this genus is more abundant in oligoasthenospermic than in healthy ejaculates (8).

In livestock species, very few papers have studied semen microbiota by massive genome sequencing technologies. Thus, it is difficult to conclude the effect of semen microbiota on fertility. To the best of our knowledge, the microbiota in goat buck semen has not yet been studied by these new technologies with very few published papers on other species. Similar to human semen, the dominant phyla in rams, rabbits, and pigs are Proteobacteria and Firmicutes, although the relative amount of these phyla varies (31.2–57.5%), depending on the species [in rams, (9); in rabbits, (10); in pigs, (11, 12)]. Actinobacteria phylum is also present in all the species (relative abundance also varies between 3.4 and 22%). Bacteroidetes are the first, third, and fourth most abundant phyla in stallions (13), pigs (11), and rabbits (10), respectively. Other reported phyla are Deinococcus-Thermus in rams (9), Fusobacteria in rabbits (10) and stallions (13), and Spirochaetes in stallions (13). The dominant genera are more variable between species. Hence, the main genera in rams are *Corynebacterium* (11%), *Pseudomonas* (10%), and *Lactococcus* (6%), while *Cupriavidus, Thermus*, and *Stenotrophomonas* represent around 5% (9). In ewes, four genera are found to play a significant role in artificial insemination (AI) success, i.e., *Mageebacillus, Histophilus, Actinobacilllus*, and *Sneathia*, because their high relative abundances in vaginal samples have clearly been associated with AI failure. The authors did not find any relation between semen microbiota and reproductive success but observed that foreskin samples of natural mating rams present these four genera. It seems plausible that the venereal transmission of these bacteria occurs during natural mating. In bulls, the most frequent genera are *Porphyromonas, Fusobacterium*, and *Ruminococcaceae UCG-010*, and the genera *W5053* and *Lawsonella* are enriched in the low-fertility group of bulls (14). In rabbits, the genera *Lysinibacillus* and *Flavobacterium* can be potential biomarkers of fertility (10). In stallions, the most frequently seen genera are *Porphyromonas* spp., *Corynebacterium* spp., and *Finegoldia* spp. (15). In pigs, the genera *Acinetobacter, Stenotrophomonas*, and *Rhodobacter* vary between ejaculates of different qualities (12).

In temperate regions, reproduction in goats is described as seasonal with major differences in seasonality between breeds and locations (16, 17). Reproductive seasonality timing is controlled by the photoperiod (18). However, the production of semen doses in goat breeding is important for the deseasonalization of meat and milk production so that supply and prices remain constant all year long (19). Many studies have confirmed that season can affect the quality characteristics of goat semen (20–24), but variation in microbiota in goat semen for different seasons has not yet been studied. A recent study published on a nonseasonal species has shown the importance of season for semen microbiota. Thus, semen microbiota in boars differs between summer and winter by presenting higher bacterial diversity in winter than in summer. Indeed, the highly abundant *Lactobacillus* genus in winter samples is positively associated with sperm quality and reproductive performance, and the highly abundant *Pseudomonas* genus in summer samples is negatively associated with sperm quality and reproductive potential (11). If season affects microbiota in nonseasonal species, it seems likely that seasonality will affect semen microbiota in seasonal breeders, and microbiota will differ in breeding and non-breeding seasons.

This study aims to characterize, for the first time, the semen microbiota in goat bucks from the Murciano-Granadina breed by the amplification and sequencing of hypervariable regions V3 and V4 of the 16S rRNA subunit gene. It studies the bacterial composition in the semen collected during the breeding and non-breeding seasons to determine whether bacterial communities can be influenced by seasonal variation.

## Materials and Methods

### Experimental Design

For the analyses of the semen microbiota and sperm quality during breeding and non-breeding seasons, the ejaculates from the same males were collected in both seasons. Two replicates per male and season were performed on different days. The semen collection during the breeding season was performed between November 26 and December 3 in 2020. During the non-breeding season, ejaculates were collected at the end of March (from 24 to 31) 2021.

#### Materials, Animals, Semen Collection, and Sample Preparation

All the chemicals were reagent grade and purchased from Sigma-Aldrich (Madrid, Spain), except for Mitotracker deep red FM, which was supplied by Invitrogen (Barcelona, Spain). Tris-citrate-glucose (TCG) diluent and TCG supplemented with bovine serum albumin (3 mg/mL; TCG-BSA) were employed to dilute semen and to perform analyses in the laboratory; NaCl solution was used to determine sperm concentration. A skimmed milk-based diluent (SM) was utilized to dilute ejaculates until they were analyzed. Their composition is described in Mocé et al. (25).

Six adult goat bucks from the Murciano-Granadina breed, housed in the Centro de Tecnología Animal, Instituto Valenciano de Investigaciones Agrarias (CITA-IVIA), were used as donors. This center is located in the Spanish town of Segorbe (Castellón; coordinates 39.86 N, 0.50 W), which belongs to the Valencian Community (east Spain). This breed presents low reproductive seasonality, and its non-breeding season goes from February to May (26). The animals were housed in pens. They were fed straw and lucerne, and a daily complement of 1 kg concentrated feed (17% crude protein, 4.5% crude oils and fat, and 11.6% crude fiber) per male. Freshwater was provided *ad libitum*. The animal housing, care, and protocols for semen collection were approved by the Animal Care and Use Committee of the IVIA and met European regulations for the care and use of animals for scientific purposes (27).

Semen was collected between 8:30 a.m. and 9:30 a.m., following the recommendations of Silvestre et al. (28). Semen collections were performed two times weekly all year long. The ejaculates were transferred to a water bath at 25°C until further processing took place. Semen volume and concentration were measured following the protocol described by Mocé et al. (25).

For the semen microbiota analyses, 0.25 mL of each ejaculate was taken and loaded into 0.25 mL plastic straws (IMV Technologies, L'Aigle, France), which were sealed with polyvinyl alcohol (PVA, IMV Technologies, L'Aigle, France). The straws were immediately stored in a freezer at −80°C until the microbiota analyses were performed. In the remaining ejaculate, the concentration was adjusted to 560 x 10^6^ sperm/mL with SM (~22°C). These samples were subsequently used to perform the sperm quality analyses.

### Sperm Quality Evaluation

Motility, sperm plasma membrane integrity (PMI), acrosomal membrane integrity, and mitochondrial functionality were evaluated in fresh samples to determine sperm quality. These analyses were performed according to the protocols described in detail in Mocé et al. (25). Briefly, all the manipulations were done at room temperature (~22°C). Motility was determined by a computer-assisted sperm analysis system (CASA; ISAS, version 1.0.17, Proiser, Valencia, Spain). Sperm motility was assessed at 37°C, with a 10 X negative phase contrast objective on a Nikon Eclipse 90i microscope (Nikon Corporation Instruments Company, IZASA, Barcelona, Spain) connected to a computer by a monochrome Basler A312f video camera (Basler Vision Technologies, Proiser, Paterna, Valencia, Spain). For each sample, the sperm concentration was adjusted with Tris-BSA (0.3%) to 6 x 10^6^ sperm/mL. The samples were incubated at 37°C for 10 min prior to evaluations. Subsamples of 7.5 μL were placed inside a Makler chamber (Counting Chamber Makler, Sefi-Medical Instruments, Haifa, Israel), prewarmed at 37°C on a thermal plate. The data from a minimum of 200 sperm from three different fields were collected. Individual sperm tracks were visually assessed to eliminate possible debris and wrong tracks. The following variables were considered in the results: proportions of total (TM; %) and progressively motile (PM; %) sperm, average path velocity (VAP; μm/s), curvilinear velocity (VCL, μm/s), straight-line velocity (VSL; μm/s), straightness index (STR; %), linearity (LIN; %), wobble (WOB; %), the amplitude of the lateral movement of the head (ALH, μm), and beat cross frequency (BCF; Hz).

Plasma membrane integrity, acrosomal membrane integrity, and mitochondria functionality were determined in each sample using flow cytometry and quadruple staining with Hoechst 33342, propidium iodide (PI), fluorescein isothiocyanate-conjugated peanut agglutinin (FITC-PNA), and Mitotracker deep red FM. The samples were stained for the flow cytometric analysis by transferring 0.1 mL aliquots with 3 x 10^6^ sperm to tubes, containing 25 μL of TCG diluent, 5 μL of Hoechst (0.1 mg/mL stock solution in Milli-Q water), and 0.25 μL of Mitotracker (25-μM stock solution in DMSO). The samples were incubated for 20 min at room temperature in the dark. Then, a solution containing 25 μL of TCG diluent with 0.25 μL of PI (1 mg/mL stock solution in Milli-Q water) and 0.5 μL of FITC-PNA (1 mg/mL stock solution in Milli-Q water) was added. The samples were incubated for another 10 min period before being diluted with 0.40 mL of TCG and analyzed by a CytoFLEX S flow cytometer (Beckman Coulter Life Sciences, L'Hospitalet de Llobregat, Barcelona, Spain), equipped with three lasers (a 50 mW 488 nm blue laser, a 50 mW 638 nm red diode laser, and an 80 mW 405 nm violet laser) and the CytExpert software (Beckman Coulter Life Sciences, L'Hospitalet de Llobregat, Barcelona, Spain). Hoechst was excited with the violet laser, and its fluorescence was detected using a 450/45 nm avalanche photodiode (APD). Mitotracker was excited with the red laser, and its fluorescence was detected, employing a 660/20 nm APD. PI and FITC-PNA were excited with the blue laser. The red fluorescence of PI was detected using a 690/50 nm APD, and the green fluorescence of FITC-PNA was detected, employing a 525/40 nm APD. Next, 50,000 events per sample were analyzed. The compensation between PI-FITC-PNA was 0.93% and was 1.39% between PI-Mitotracker. NonDNA-containing events (Hoechst negative) were excluded, and the sperm population was gated based on the expected forward and side scatter signals (29). The PI penetrated non-viable cells to distinguish three populations: PI– (plasma membrane intact sperm), and PI+ with high (dead sperm) or low fluorescence intensity (apoptotic sperm). Only the sperm with damaged acrosomes stained with FITC-PNA and two populations were distinguished (FITC-PNA+ and FITC-PNA–). Finally, all the sperm stained with Mitotracker and two populations were distinguished: one with low-intensity corresponding to the sperm with a low mitochondria membrane potential (MMP) and another with high intensity corresponding to the sperm with high MMP (30). First spermatozoa were categorized according to stains PI and FITC-PNA as plasma membrane intact with acrosome intact (PI–/FITC-PNA–), reacted (PI–/FITC-PNA+) or plasma membrane damaged with acrosome intact (PI+/FITC-PNA–) or reacted (PI+/FITC-PNA+) sperm. The sperm populations that exhibited intact plasma membrane (PMI; PI–), apoptotic sperm (PI+ with low intensity), acrosome-reacted sperm (AR; FITC-PNA+), and PMI acrosome-intact sperm (PMI-AI; PI–/FITC-PNA–) were reported. PI and Mitotracker were also plotted on another chart, and four sperm populations were obtained: plasma membrane intact with low MMP (PI–/low MMP) or high MMP (PI–/high MMP) or plasma membrane damaged with low MMP (PI+/low MMP) or high MMP (PI+/high MMP). From this chart, the sperm populations that exhibited high MMP and PMI (PI–) with high or low MMP were reported. Finally, only for the population of plasma membrane intact with acrosome intact (PI–/FITC-PNA–) were the proportions of sperm with low or high MMP calculated.

### Microbiota Analyses

#### DNA Extraction, PCR Amplification, Library Preparation, and Sequencing

Total DNA was extracted using the DNeasy PowerLyzer PowerSoil Kit (Qiagen, Hilden, Germany), following the manufacturer's instructions. Mock community DNA was included as a positive control for library preparation (Zymobiomics Microbial Community DNA, ZymoResearch, Irvine, CA, USA). PCR amplification of the bacterial 16S rRNA gene V3–V4 regions was performed with the forward and the reverse primers (5′-TCGTCGGCAGCGTCAGATGTGTATAAGAGACAGCCTACGGGNGGCWGCAG-3′) and (5′-GTCTCGTGGGCTCGGAGATGTGTATAAGAGACAGGACTACHVGGGTATCTAATCC-3′). The PCR conditions consisted of an initial 95°C for 3 min (initial denaturation), followed by 30 cycles: 30 s at 95°C, 30 s at 55°C, and 30 s at 72°C, and a final extension step of 5 min at 72°C. Libraries were normalized using the SequalPrep Normalization Plate Kit (Applied Biosystems, ThermoFisher Scientific, Waltham, MA, USA) and then pooled. The final pools were purified with AMPure XP beads (Beckman Coulter, Nyon, Switzerland) and quantified with the KAPA library quantification kit for Illumina Platforms (Kapa Biosystems, SigmaAldrich, Saint Louis, MO, USA) on an ABI 7900HT real-time cycler (Applied Biosystems, ThermoFisher Scientific, Waltham, MA, USA). Sequencing was carried out using the Illumina MiSeq platform with 2 x 300 bp reads. Negative controls were analyzed to detect any environmental/kit-derived contaminant amplicons.

#### Bacterial 16S rRNA Sequences Processing

The raw demultiplexed forward and reverse reads were processed using the Quantitative Insights into Microbial Ecology (QIIME2, version 2019.4) software (31). Quality filtering, denoizing, pair-end merging, and amplicon sequence variant calling (ASV, i.e., phylotypes or Operational Taxonomic Units, OTUs) were carried out with the DADA2 pipeline incorporated into QIIME2 (32).

The reads were truncated at the position when the 75th percentile Phred score fell below Q20: 300 bp for the forward reads and 251 bp for the reverse reads.

Alpha diversity metrics, measured as observed OTUs (i.e., community richness), Pielou's evenness index (community evenness), and Shannon's diversity index (community richness), were analyzed to investigate semen microbiota diversity. Beta diversity analysis was carried out to study the structural variation of microbial communities across samples using unweighted and weighted Unifrac distances. The visualization of microbial communities' structure was done with principal coordinate analysis (PCoA) plots.

### Statistical Analyses

The statistical model used to analyze the sperm quality variables included the fixed effects of replicate (two levels), season (breeding and non-breeding seasons), and the interaction between both factors. Male was included as a random effect. For those variables not following normal probability distribution (LIN, STR, WOB, BCF, AR, PMI- low MMP, and PMI-AI with high MMP), a nonparametric test was used (Wilcoxon test) to first test the differences between replicates and then to test the differences between seasons. Statistical analyses were run using SPSS® 27.0 (IBM Corporation, New York, NY, USA). The level of significance was set at *p* < 0.05.

Alpha diversity comparisons were made by a robust two-way ANOVA (33). Differences in beta diversity were assessed by the PERMANOVA test. The differential relative abundance of taxa was tested by a robust two-way ANOVA analysis. The model used to analyze alpha and beta diversity and the differential relative abundance of taxa included the fixed effects of the replicate (two levels), season (breeding and non-breeding seasons), and the interaction between both factors. Spearman correlation coefficients were calculated to study the relationship between the semen quality parameters that differed between seasons (VCL, VSL, VAP, LIN, STR, WOB, ALH, apoptotic sperm, plasma membrane intact sperm with a low mitochondria membrane potential; plasma membrane and acrosome-intact sperm with a high mitochondria membrane potential) and the mean relative abundance of those bacteria that differed between seasons at the genus level (*Sphingomonas, Halomonas, Methanobrevibacter*, and *Faecalibacterium*). Statistical analyses were run using R Packages (BiodiversityR version 2.11-1, PMCMR version 4.3, RVAideMemoire version.9-7, vegan version 2.5-5 packages, and WRS2 version 1.1-3). The level of significance was set at *p* < 0.05.

## Results

### Sperm Quality

The replicate and interaction effects were not significant for any studied variable. Table 1 shows the values for the goat buck's fresh sperm quality variables during the breeding and non-breeding seasons. No differences were found for total motile sperm or progressively motile sperm between seasons. The concentration was higher (*p* < 0.05) for the non-breeding than for the breeding season, but semen production was similar during both seasons (3,059 and 2,685 x 10^6^ sperm). Seasons affected mainly sperm movement quality. The ejaculates collected during the breeding season exhibited higher velocities (VCL, VSL, and VAP) and velocity indices (LIN, STR, and WOB), and lower ALH values than the ejaculates collected during the non-breeding season (*p* < 0.05). The ejaculates from both seasons also differed in the proportions of both apoptotic spermatozoa and the plasma membrane intact sperm with low MMP, which were higher during the non-breeding season, and also in the proportion of plasma membrane and acrosome-intact sperm with high MMP, which was lower for the non-breeding than for the breeding season (*p* < 0.05).

**Table 1:** Quality of the fresh goat buck ejaculates collected during the non-breeding and breeding seasons.

	**Non-breeding season**	**Breeding season**	***p***
Concentration (x10^6^ sperm/mL)	3,214 ± 193	2,609 ± 193	^*^
Volume (mL)	0.9 ± 0.13	1.0 ± 0.13	
Production (x10^6^ sperm)	3,059 ± 493	2,685 ± 493	
**CASA system**			
TM (%)	60 ± 4.6	66 ± 4.6	
PM (%)	41 ± 4.2	54 ± 4.2	
VCL (μm/s)	119 ± 2.6	128 ± 2.6	^*^
VSL (μm/s)	85 ± 4.6	110 ± 4.6	^*^
VAP (μm/s)	98 ± 3.8	119 ± 3.8	^*^
ALH (μm)	2.6 ± 0.11	1.9 ± 0.11	^*^
LIN (%)^a^	69 ± 2.7	82 ± 2.7	^*^
STR (%)^a^	80 ± 1.9	88 ± 1.9	^*^
WOB (%)^a^	82 ± 1.7	91 ± 1.7	^*^
BCF (Hz)^a^	11.2 ± 0.23	10.9 ± 0.23	
**Cytometer**			
PMI (%)	56 ± 3.7	57 ± 3.7	
Apoptotic sperm (%)	9.7 ± 0.68	6.9 ± 0.68	^*^
AR (%)^a^	12 ± 2.0	13 ± 2.0	
PMI-AI (%)	56 ± 3.7	57 ± 3.7	
High MMP (%)	69 ± 4.1	72 ± 4.1	
PMI- high MMP (%)	55 ± 3.6	56 ± 3.6	
PMI- low MMP (%)^a^	1.4 ± 0.23	0.6 ± 0.23	^*^
PMI-AI with high MMP (%)^a^	97.2 ± 0.48	98.7 ± 0.48	^*^

a *Indicates that variables were analyzed by a nonparametric test*.

* *Indicates significant differences (p < 0.05)*.

*TM, total motile sperm; PM, progressively motile sperm; VCL, curvilinear velocity; VSL, straight line velocity; VAP, average path velocity; ALH, amplitude of the lateral head movement; LIN, linearity index; STR, straightness index; WOB, wobble; BCF, beat cross frequency; PMI, plasma membrane intact sperm [propidium iodide negative (PI-)]; apoptotic sperm, PI+ with low intensity; AR, acrosome-reacted sperm (FITC-PNA+); PMI-AI, plasma membrane and acrosome intact sperm (PI-/FITC-PNA-); high MMP, sperm with a high mitochondria membrane potential (high intensity of Mitotracker deep red FM); PMI- high MMP, plasma membrane intact sperm (PI-) with a high mitochondria membrane potential; PMI- a low MMP, plasma membrane intact sperm (PI-) with a low mitochondria membrane potential; PMI-AI with high MMP (%), plasma membrane and acrosome-intact sperm (PI-/FITC-PNA-) with a high mitochondria membrane potential*.

### Semen Microbiota

To describe the microbiota in goat buck ejaculates and to determine if it differed during the breeding and non-breeding seasons, microbial genomic DNA was isolated from 24 ejaculates.

#### Sequencing Overview

In all, 3,785,779 pair-end reads were obtained, and 3,045,948 reads remained after the quality filtering, trimming, and denoizing steps. Paired-end reads were merged. After chimera removal, 2,066,478 merged reads were used for phylotype calling with DADA2 (32). After quality control, 1,688 phylotypes were detected. Singletons and doubletons were removed before the diversity analysis.

#### Diversity Analysis

##### Alpha Diversity

**Figure 1:**
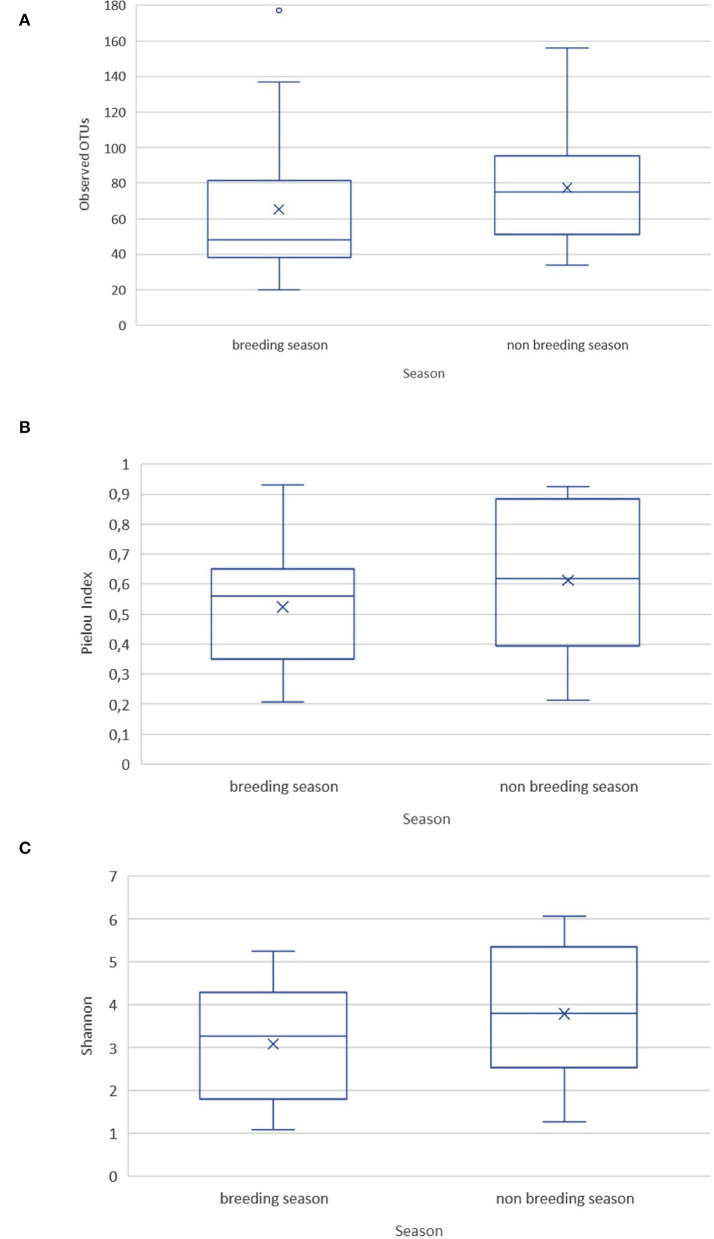
Alpha diversity metrics for the goat buck ejaculates collected during the breeding and non-breeding seasons. **(A)** Observed OTUs; **(B)** Pielou's evenness index; **(C)** Shannon's diversity index.

Rarefaction curves showed that the depth of sequencing and the achieved subsampling size were sufficient to observe the complete diversity present in the sampled microbial communities. A plateau was reached for all the calculated alpha diversity metrics (Supplementary Figure 1). The alpha diversity metrics were measured as observed OTUs (i.e., community richness), Pielou's evenness index (community evenness), and Shannon's diversity index (community richness). They showed no significant difference between the ejaculates collected during the breeding and non-breeding season (Figure 1), or between replicates within a season.

##### Beta Diversity

**Figure 2:**
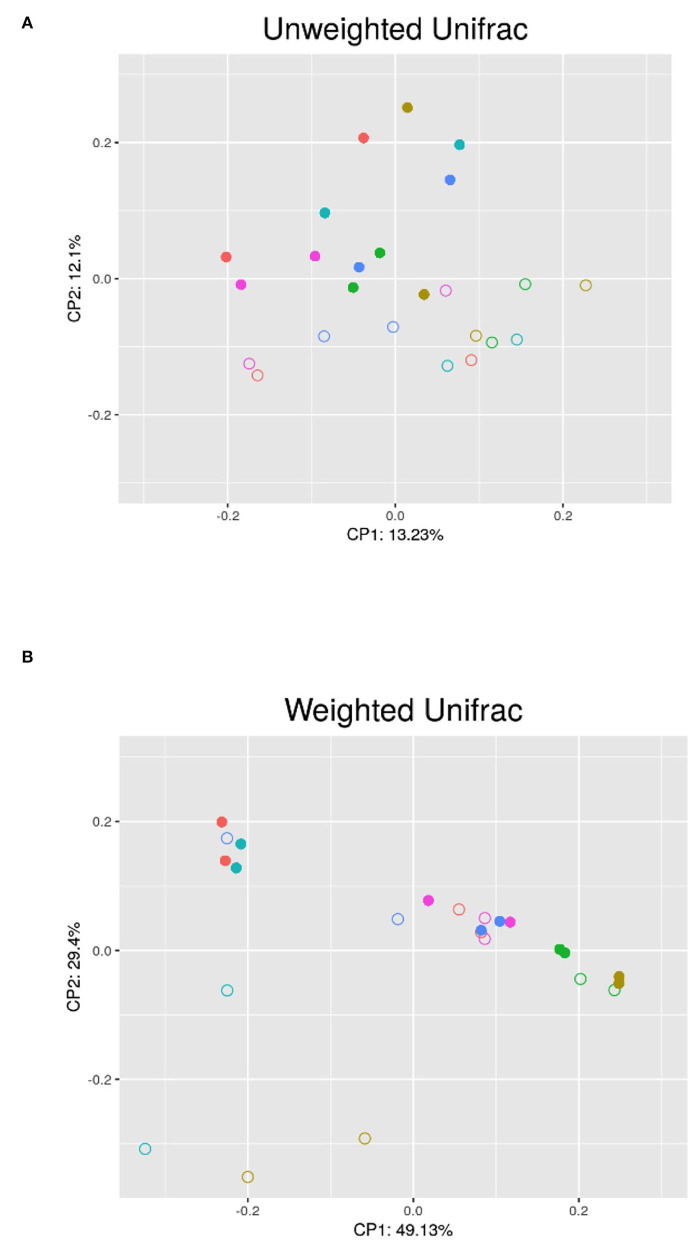
PCoA analysis of the semen microbiota in the different bucks (each color belongs to a different buck) between the non-breeding (•) and breeding (°) seasons; **(A)** Unweighted UniFrac PCoA; **(B)** Weighted UniFrac PCoA; CP1 and CP2: first and second principal coordinates.

The results of the PERMANOVA test using unweighted and weighted UniFrac distances showed significant differences in the microbial community structure (*p* < 0.05) between the breeding and non-breeding season (Figure 2). The replicate and the interaction between replicate and seasons did not affect beta diversity.

#### Taxonomic Composition of Goat Buck Semen Microbiota During the Breeding and Non-breeding Seasons

Archaeal communities were detected in 58% (14/24) of the semen samples at low mean relative abundance (0.21%). Three phyla were detected (Euryarchaeota, Halobacterota, and Thermoplasmatota). Of the three, the phyla Halobacterota and Thermoplasmatota were detected only in one sample and two samples, respectively.

**Figure 3:**
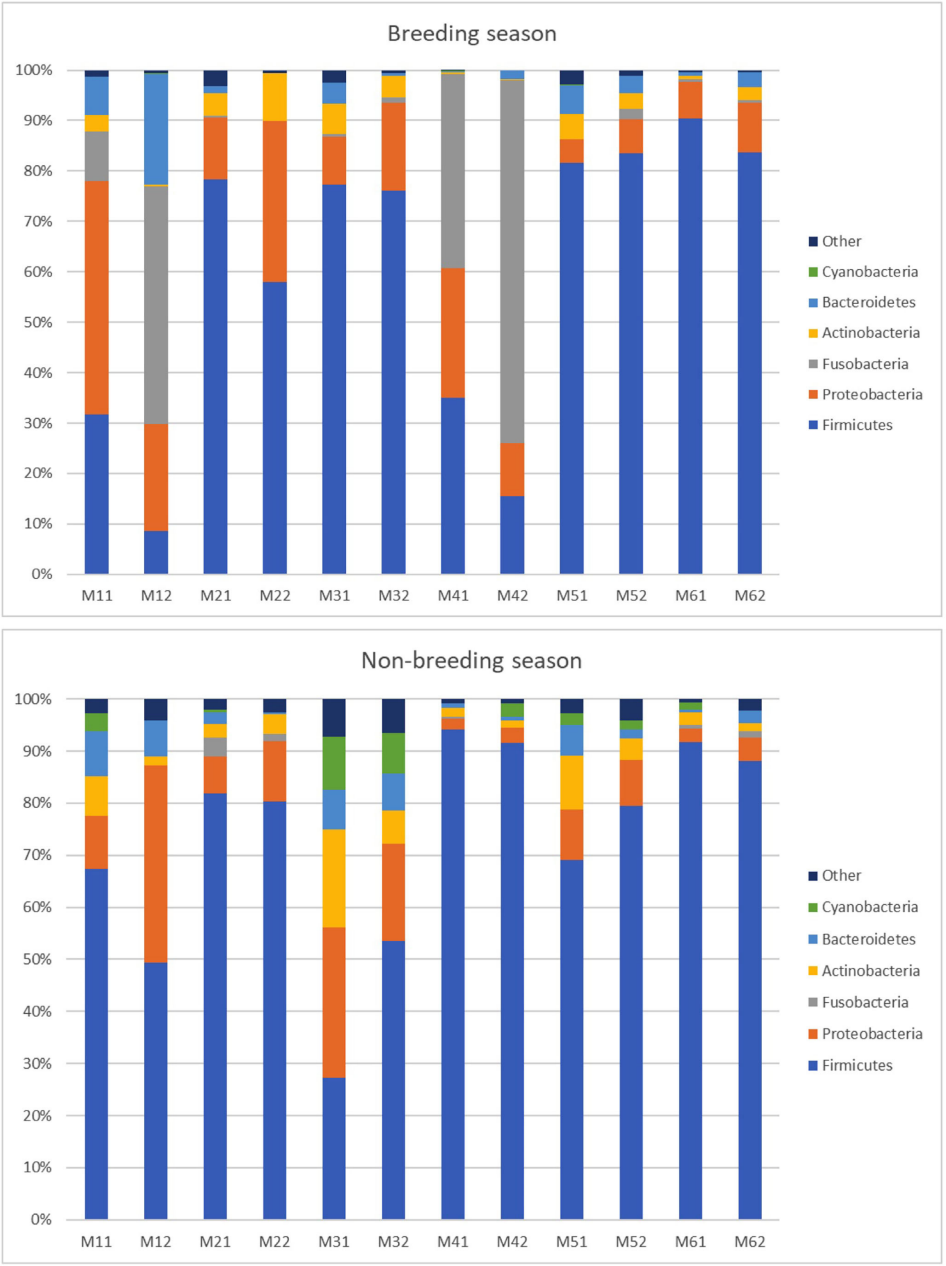
Relative abundance of taxa at the phylum level in the individual goat buck ejaculates collected during the breeding and non-breeding seasons (only the taxa with a mean relative abundance > 1.5% for the breeding or non-breeding seasons are represented). The letters on the x-axis correspond to each individual sample. M11 and M12 are the samples of Male 1; M21 and M22 are the samples of Male 2; M31 and M32 are the samples of Male 3; M41 and M42 are the samples of Male 4; M51 and M52 are the samples of Male 5; and M61 and M62 are the samples of Male 6.

Regarding bacterial communities, 19 bacterial phyla in the ejaculates from the breeding season and 23 bacterial phyla from the non-breeding season were identified from the 24 detected phyla. This means that 18 phyla were coincident during both seasons; one appeared only during the breeding season and five only during the non-breeding season. The phyla that appeared only during one of the seasons were observed at the most in one or two of the sampled animals. Figure 3 shows that the five dominant phyla in the ejaculates collected during the breeding season were Firmicutes (59.98%), Proteobacteria (16.97%), Fusobacteria (14.34%), Actinobacteria (3.34%), and Bacteroidetes (4.22%). The most abundant phyla during the non-breeding season were Firmicutes (72.81%) Proteobacteria (12.05%), Actinobacteria (5.24%), Bacteroidetes (3.75%), and Cyanobacteria (2.48%). The composition of microbial communities was variable between animals. Firmicutes were the most abundant phylum but ranged between 92% (measure M6.1) and 9% (measure M1.2). For the phylum Fusobacteria, two males presented higher proportions (28% for Male 1 and 55% for Male 4). A robust two-way ANOVA analysis showed decreased relative abundance for phyla Planctomycetota and Cyanobacteria during the breeding season (0.05% vs. 0.66% for Planctomycetota and 0.03 vs. 2.48 for Cyanobacteria; *p* < 0.05) and an increase in the domain Archaea phylum Euryarchaeota (0.28 vs. 0.09; *p* < 0.05).

**Figure 4:**
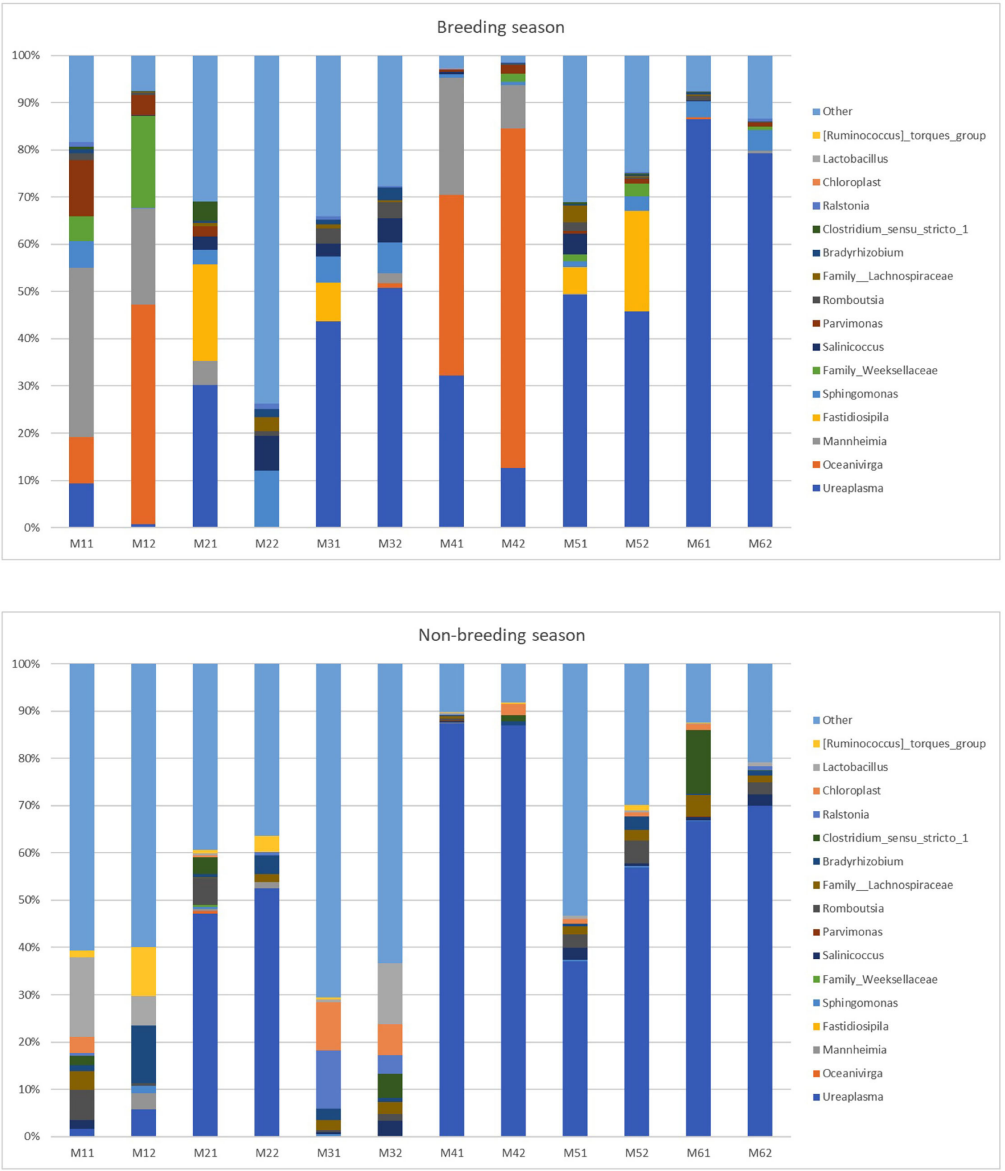
Relative abundance of taxa at the genus level in the individual goat buck ejaculates collected during the breeding and non-breeding seasons (only the taxa with a mean relative abundance >1.5% for the breeding or non-breeding seasons are represented). The letters on the x-axis correspond to each individual sample. M11 and M12 are the samples of Male 1; M21 and M22 are the samples of Male 2; M31 and M32 are the samples of Male 3; M41 and M42 are the samples of Male 4; M51 and M52 are the samples of Male 5; and M61 and M62 are the samples of Male 6.

At the genus level (or the family level when genus could not be assigned), 424 taxa were identified (235 for the breeding season; 346 for the non-breeding season). Nine genera and one family during the breeding season and 11 genera and two families during the non-breeding season showed relative abundance >1% (Figure 4 and Table 2). This means that the relative abundance for most taxa was low. The dominant microbial genera were *Ureaplasma* (36.71%), *Oceanivirga* (13.99%), and *Mannheimia* (8.18%) during the breeding season and *Ureaplasma* (42.67%), *Lactobacillus* (3.28%), and *Bradyrhizobium* (2.25%) during the non-breeding season (Figure 3 and Table 2). A robust two-way ANOVA analysis showed a decrease in the relative abundance of the genus *Faecalibacterium* during the breeding season (0.20 vs. 1%, *p* < 0.05) and an increase in the genera *Sphingomonas* and *Halomonas* (3.88 vs. 0.29% and 1.09 vs. 0.20% for *Sphingomonas* and *Halomonas*, respectively, *p* < 0.05). Our results also showed differences in the archaeal community, with an increase in the *Methanobrevibacter* genus during the breeding season (0.27 vs. 0.09, *p* < 0.05). Spearman correlations (Table 3) showed that the genus *Sphingomonas* correlated positively with VSL, LIN, WOB, and the sperm population exhibiting plasma membrane and acrosome intact and a high mitochondria membrane potential. This genus also correlated negatively with ALH and the sperm population presenting an intact plasma membrane and a low mitochondrial membrane potential. The amplitude of lateral head movement also correlated negatively with *Halomonas* abundance. The genus *Methanobrevibacter* correlated negatively with the proportion of plasma membrane intact sperm with a low mitochondria membrane potential and positively with the percentage of plasma membrane and acrosome-intact sperm with a high MMP. The genus *Faecalibacterium* correlated negatively with LIN, STR, and WOB, and positively with ALH.

**Table 2:** Mean relative abundance (%) of the taxa at the genus level (or the family level when the genus could not be assigned) in the goat buck ejaculates collected during the breeding and non-breeding seasons.

**Taxa**	**Breeding season**	**Non-breeding season**
*Ureaplasma*	36.71	42.67
*Oceanivirga*	13.99	0.04
*Mannheimia*	8.18	0.45
*Fastidiosipila*	4.63	0.00
*Sphingomonas*	3.88	0.29
*Lactobacillus*	0.00	3.28
*f_Weeksellaceae*	2.60	0.03
*Bradyrhizobium*	0.66	2.25
*Chloroplast*	0.02	2.18
*Clostridium_sensu_stricto_1*	0.47	2.13
*Romboutsia*	1.12	2.11
*Salinicoccus*	1.93	1.00
*Parvimonas*	1.92	0.03
*f_Lachnospiraceae*	0.75	1.72
*Ralstonia*	0.32	1.55
*[Ruminococcus]_torques_group*	0.00	1.51
*f__Peptostreptococcaceae_*	0.74	1.42
*Cutibacterium*	0.84	1.20
*Mitochondria*	0.00	1.11
*Halomonas*	1.09	0.20
*Other*	23.95	35.86

*Only the taxa with a mean relative abundance >1% for the breeding or non-breeding seasons are shown. The other category includes those genera with a mean relative abundance <1%*.

**Table 3:** Spearman's rank correlation coefficients between the mean relative abundance of the genera *Sphingomonas, Halomonas, Methanobrevibacter*, and *Faecalibacterium* and the sperm quality parameters of goat buck ejaculates.

	***Sphingomonas***	***Halomonas***	***Methanobrevibacter***	***Faecalibacterium***
VCL (μm/s)	0.189	0.003	0.102	0.003
VSL (μm/s)	0.411^*^	0.244	0.197	−0.371
VAP (μm/s)	0.375	0.206	0.195	−0.330
LIN (%)	0.459^*^	0.340	0.190	−0.555^*^
STR (%)	0.346	0.357	0.078	−0.482^*^
WOB (%)	0.442^*^	0.312	0.202	−0.551^*^
ALH (μm)	−0.464^*^	−0.416^*^	−0.194	0.567^*^
Apoptotic sperm (%)	−0.261	−0.288	0.194	0.380
PMI- a low MMP (%)	−0.637^*^	−0.384	−0.681^**^	0.217
PMI-AI with a high MMP (%)	0.579^*^	0.356	0.734^**^	−0.134

* *Indicates that the correlation coefficient significantly differs from zero (p < 0.05)*.

*VCL, curvilinear velocity; VSL, straight line velocity; VAP, average path velocity; LIN, linearity index; STR, straightness index; WOB, wobble; ALH, amplitude of lateral head movement; apoptotic sperm, PI+ with low intensity; PMI- low MMP: plasma membrane intact sperm (PI-) with a low mitochondria membrane potential; PMI-AI with a high MMP, plasma membrane and acrosome-intact sperm (PI-/FITC-PNA-) with a high mitochondria membrane potential*.

## Discussion

Variations in the microbiota of ejaculated goat buck semen during different seasons are currently unknown. This study characterizes, for the first time, the semen microbiota in Murciano-Granadina goat bucks by sequencing hypervariable regions V3 and V4 of the 16S rRNA subunit gene and studying bacterial semen diversity during different seasons. Unlike studies based on culture-dependent methods, the studies based on metagenomics allow microbiota complexity to be captured and can identify large numbers of the bacteria present in a sample (2, 3, 14, 15).

Murciano-Granadina is a breed with low reproductive seasonality. To confirm the differences in sperm quality between the breeding and non-breeding seasons, we first evaluated the differences in sperm motility, kinematics, and the status of the plasma membrane, acrosome, and mitochondria functionality. Seasonality mostly affected sperm movement quality (Table 1). All the velocities (VCL, VSL, VAP) and the ratios of the three velocities (LIN, STR, and WOB) were higher during the breeding than during the non-breeding season. Moreover, the amplitude of lateral head movement (ALH) was higher during the non-breeding season. These results showed that the sperm collected during the breeding season showed more regular and linear trajectories with less lateral movement than the sperm collected during the non-breeding season. In addition, the proportion of apoptotic sperm was slightly higher for the non-breeding season, as was the proportion of the plasma membrane intact sperm with low mitochondrial membrane potential. Murciano–Granadina is a Spanish goat breed that is well-adapted to Mediterranean environmental conditions. Previous studies have shown significant seasonal variation in semen quantity and quality, but we can consider that it is a breed with low reproductive seasonality (21, 22, 26, 34). Although the males from this breed show a longer reaction time in spring (34), and plasma testosterone concentrations display a well-defined seasonal pattern (26, 35), the quality of ejaculates is good all year long (21).

We found that goat semen contained a high diversity of bacteria. The five observed dominant phyla (Firmicutes, 66.39%; Proteobacteria, 14.51%; Fusobacteria, 7.47%; Actinobacteria, 4.29%; Bacteroidetes, 3.98%) coincided with those phyla usually reported in other livestock species like rabbits (10), pigs (11, 12), stallions (13), rams (9) and bovine (36), although the relative abundance of the different taxa varied between species. Thus, for example, the phylum Proteobacteria ranged between 4.3% in stallions (13) and 57% in pigs (11), and the phylum Fusobacteria went from 22% in rabbits (10) to 0.2% in rams (9). In our study, Firmicutes was the most abundant phylum, but a high diversity between the samples was observed (Figure 3). For the phylum Fusobacteria, two males presented a very high proportion, but this phylum was almost absent in the other males. Several studies in humans and stallions have shown that semen bacterial communities contain diverse taxa, which vary considerably among individuals (4, 5, 7, 13). Previous studies have sampled semen at a single time point, but microbiota stability within a season has not been studied (37). Our results demonstrate that, while microbiota varied between seasons according to beta diversity, it remained stable within a season (the microbiota composition of ejaculates was similar in the two replicates of the same male for the same season). The time between replicates ranged from 2 to 7 days, which indicates that microbiota composition remained stable for 7 days.

Regarding genera, the relative abundances of taxa at the genus level in studies published to date are variable. Zhang et al. (11) reported that the most abundant genera in pigs are *Pseudomonas, Lactobacillus*, and *Ralstonia* with mean relative abundances of 34.41, 19.93, and 6.82%, respectively; Serrano et al. (9) indicated that the most abundant genera in rams are *Corynebacterium* (11%), *Pseudomonas* (10%), and *Lactococcus* (6%). The most abundant genera in bulls are *Porphyromonas, Fusobacterium, Ruminococcaceae*, and *UCG-010* (14). In stallions, the most frequently seen genera are *Porphyromonas* spp., *Corynebacterium* spp., and *Finegoldia* spp. (15).

In our study, *Ureaplasma* was the most abundant genus for both seasons. In rams, this genus was among the 10 most abundant genera, but its abundance was lower (3%) (9). Although *Ureaplasma diversum* has been associated with bovine reproductive illnesses (38, 39), recent studies have reported that *Ureaplasma* is the most abundant genus (~33%) in the vaginal microbiota of healthy dairy heifers that do not exhibit visible vulvar lesions, with mean fertility of 85% upon first insemination (40). Moreover, in ovids, the *Ureaplasma* genus does not seem to affect AI fertility (9). Thus, more studies are necessary to unravel the effect of *Ureaplasma* on reproduction.

*Pseudomonas* and *Corynebacterium*, which were the two most abundant genera in rams (9), were also present in all the males in our study but in smaller quantities (0.55 and 0.73%, respectively). *Corynebacterium* has also been observed in the semen of bulls (14) and stallions (15). *Corynebacterium* is distributed in soil and water and colonizes animal skin and mucous membranes (14). Attention should be paid to the presence of *Pseudomonas* because the venereal transmission of this bacterium has been previously confirmed in horses (41) to provoke uterine disease.

In our study, the *Mannheimia* genus was observed during the breeding season with a mean relative abundance of 8.17%. This genus has also been detected in the semen of one of the five rams, and the vagina of 10 of the 50 ewes tested in the work of Serrano et al. (9). In small ruminants, *Mannheimia haemolytica* appears as natural flora in the upper respiratory system and is considered an opportunistic pathogen in severe pleuropneumonias in cattle, sheep, and goats (42). The presence of typical bacteria from the respiratory tract in semen has been reported in previous studies [*Pseudomonas aeruginosa* and *Klebsiella pneumonia* in stallions; (15)]. It is unknown how the presence of bacteria considered opportunistic can affect sperm quality and fertility, especially in samples that need to be stored before insemination (15).

The genus *Oceanivirga* appeared mostly in the goat ejaculates collected during the breeding season and has also been detected in mare vagina (43, 44) and bull semen (14). This genus belongs to the *Leptotrichiaceae* family, which typically colonizes human or animal oropharynx, respiratory, urogenital, and gastrointestinal tracts (45, 46). The importance that these genera may have for reproductive performance remains unknown.

No significant differences were observed in the richness and evenness between the samples collected during the breeding and non-breeding seasons (Figure 2), but beta diversity significantly differed between both groups (Figure 3), which, thus, indicates that seasonality affected microbial community composition. We found differences in taxonomic composition between the breeding and non-breeding seasons. Our results revealed that the relative abundance of the *Faecalibacterium* genus significantly decreased during the breeding season, while the proportion of the *Sphingomonas* and *Halomonas* genera increased. Moreover, an increase in the *Methanobrevibacter* genus in the archaeal community was observed during the breeding season. According to our results, the ejaculates collected during the breeding season were characterized by a higher abundance of the genus *Sphingomonas*, and this bacterium could positively impact sperm quality based on the observed correlations (Table 3). Furthermore, the higher abundance of the genus *Faecalibacterium* during the non-breeding season could negatively impact sperm quality according to the detected correlations (Table 3). In humans, a significant reduction in *Sphingomonas* has been reported in the asthenospermia group (8), and seminal microbiota has been characterized in the oligoasthenospermia group by the dominance of several bacteria, including *Faecalibacterium* (8). Taken together, our results suggest that *Sphingomonas* and *Faecalibacterium* could be possible biomarkers of sperm quality in goat bucks. Both the *Faecalibacterium* and *Methanobrevibacter* genera are typically present in the digestive community of goats (47–49). Indeed, *Methanobrevibacter* is the most abundant genus in the goat rumen archaeal community (49). *Halomonas* has been organized as a genus since 1980, is typically found in saline environments, was originally seen as environmental contaminants (50), and has been recently observed in 68% (17 out of 25) ram foreskin samples (9). Bacterial ejaculate contamination is frequent due to the inherent nature of the semen collection process (51). Sources of bacterial contamination include, but are not limited to, the normal microflora of foreskin, feces, skin, and hair microorganisms (14, 15, 51, 52). Godià et al. (12) reported that the predominant species contaminated Pietrain boar semen after ejaculation, which came from the soil, feces, and water sources (*Bacillus megaterium, Brachybacterium faecium, Bacillus coagulans)*. In bulls and stallions, most of the detected bacteria originated from either the environment or the mucosa of animals and humans [*Porphyromonas, Fusobacterium, Fastidiosipila, Cutibacterium, Corynebacterium*; (14, 15)]. The presence of *Sphingomonas* has been previously reported in normal human semen (1, 8), detected in the colon microbiota of goats and is among the 20 most abundant OTUs in the colon (47). Although our semen collection was performed under very hygienic conditions, likely, the presence of the *Faecalibacterium, Methanobrevibacter, Halomonas* and *Sphingomonas* genera originated from contamination by environmental microorganisms.

However, the colonization of semen with these genera could occur as a result of some male behavior. Male homosexual behavior, as well as penis licking and masturbation, is common in ruminant species, but these behaviors are more frequent in goat bucks that are isolated from estrous females (53), which is the case in AI centers. Such behavior can bring about semen colonization with fecal and gut bacteria. Vaginal colonization by fecal bacteria has been proposed in heifers and mares (43, 54, 55). Finally, evidence for a possible link between the gut and seminal microbiomes has been reported in mice, where significant changes in the seminal microbiome were observed in the animals fed with a high-fat diet (56). All these phenomena could explain the presence of the typical genera from the gut in goat semen. However, the role that these genera could play in semen quality remains to be elucidated.

In conclusion, our study describes, for the first time, the semen microbiota of goat bucks by sequencing hypervariable regions V3 and V4 of the 16S rRNA subunit gene. It also shows that, while seasons affect microbial community composition, microbioma remains stable within seasons for 7 days. In addition, the genera *Sphingomonas* and *Faecalibacterium* could be possible biomarkers of semen quality in goat bucks. These results contribute to an in-depth understanding of the effects of reproductive seasonality on goat buck ejaculates.

## Data Availability Statement

The datasets presented in this study can be found in online repositories. The names of the repository/repositories and accession number(s) can be found at: https://www.ncbi.nlm.nih.gov/bioproject/789859.

## Ethics Statement

The animal study was reviewed and approved by Animal Care and Use Committee of IVIA.

## Author Contributions

MM designed the study, analyzed data, interpreted results, and wrote the original draft of the manuscript. IE and SP-F performed the sample collection and laboratory work. EG designed the study, analyzed the data, and interpreted the results. EM designed the study, coordinated the study, interpreted the results, and performed the sample collection and laboratory work. All the authors provided critical feedback, edited the manuscript, and approved the submitted version.

## Funding

This work was supported by the Instituto Nacional de Investigación y Tecnología Agraria y Alimentaria (INIA) and was cofinanced by ERDF (Grant No. RTA2017-00049-C02-01), GVA-IVIA and was co-funded by the EU through the Operational Programmes ERDF of the Comunitat Valenciana 2014-2020 and 2021-2027 (Grant Nos. 51906 and 52201K), AMURVAL (Contract No. 71714), and by the Universidad Cardenal Herrera-CEU, CEU Universities (Grant Nos. INDI20/34 and INDI21/40).

## Conflict of Interest

The authors declare that the research was conducted in the absence of any commercial or financial relationships that could be construed as a potential conflict of interest.

## Publisher's Note

All claims expressed in this article are solely those of the authors and do not necessarily represent those of their affiliated organizations, or those of the publisher, the editors and the reviewers. Any product that may be evaluated in this article, or claim that may be made by its manufacturer, is not guaranteed or endorsed by the publisher.

## References

[1] Altmäe S Franasiak JM Mändar R. The seminal microbiome in health and disease. Nat Rev Urol. (2019) 16:703–21. 10.1038/s41585-019-0250-y 31732723

[2] Koedooder R Mackens S Budding A Fares D Blockeel C Laven J. Identification and evaluation of the microbiome in the female and male reproductive tracts. Hum Reprod Update. (2019) 25:298–325. 10.1093/humupd/dmy048 30938752

[3] Lundy SD Vij SC Rezk AH Cohen JA Bajic P Ramasamy R. The microbiome of the infertile male. Curr Opin Urol. (2020) 30:355–62. 10.1097/MOU.0000000000000742 32235279

[4] Baud D Pattaroni C Vulliemoz N Castella V Marsland BJ Stojanov M. Sperm microbiota and its impact on semen parameters. Front Microbiol. (2019) 10:234. 10.3389/fmicb.2019.00234 30809218 PMC6379293

[5] Hou D Zhou X Zhong X Settles ML Herring J Wang L. Microbiota of the seminal fluid from healthy and infertile men. Fertil Steril. (2013) 100:1261–9. 10.1016/j.fertnstert.2013.07.1991 23993888 PMC3888793

[6] Monteiro C Marques PI Cavadas B Damião I Almeida V Barros N. Characterization of microbiota in male infertility cases uncovers differences in seminal hyperviscosity and oligoasthenoteratozoospermia possibly correlated with increased prevalence of infectious bacteria. Am J Reprod Immunol. (2018) 79:e12838. 10.1111/aji.12838 29500854

[7] Weng SL Chiu CM Lin FM Huang WC Liang C Yang T. Bacterial communities in semen from men of infertile couples: metagenomic sequencing reveals relationships of seminal microbiota to semen quality. PLoS ONE. (2014) 9:e110152. 10.1371/journal.pone.0110152 25340531 PMC4207690

[8] Yang H Zhang J Xue Z Zhao C Lei L Wen Y. Potential pathogenic bacteria in seminal microbiota of patients with different types of dysspermatism. Sci Rep. (2020) 10:6876. 10.1038/s41598-020-63787-x 32327694 PMC7181748

[9] Serrano M Climent E Freire F Martínez-Blanch JF González C Reyes L. Influence of the ovine genital tract microbiota on the species artificial insemination outcome. A pilot study in commercial sheep farms. High Throughput. (2020) 9:16. 10.3390/ht9030016 PMC7576495 32640606

[10] Marco-Jiménez F Borrás S García-Domínguez X D'Auria G Vicente JS Marín C. Roles of host genetics and sperm microbiota in reproductive success in healthy rabbit. Theriogenology. (2020) 158:416–23. 10.1016/j.theriogenology.2020.09.028 33039925

[11] Zhang J Liu H Yang Q Li P Wen Y Han X. Genomic sequencing reveals the diversity of seminal bacteria and relationships to reproductive potential in boar sperm. Front Microbiol. (2020) 11:1873. 10.3389/fmicb.2020.01873 32903829 PMC7438901

[12] Gòdia M Ramayo-Caldas Y Zingaretti LM Darwich L Lopez S Rodríguez-Gil JE. A pilot RNA-seq study in 40 pietrain ejaculates to characterize the porcine sperm microbiome. Theriogenology. (2020) 157:525–33. 10.1016/j.theriogenology.2020.08.001 32971422

[13] Quiñones-Pérez C Hidalgo M Ortiz I Crespo F Vega-Pla JL. Characterization of the seminal bacterial microbiome of healthy, fertile stallions using next-generation sequencing. Anim Reprod. (2021) 18:e20200052. 10.1590/1984-3143-ar2020-0052 34394753 PMC8356074

[14] Cojkic A Niazi A Guo Y Hallap T Padrik P Morrell JM. Identification of bull semen microbiome by 16S sequencing and possible relationships with fertility. Microorganisms. (2021) 9:2431. 10.3390/microorganisms9122431 34946031 PMC8705814

[15] Al-Kass Z Guo Y Vinnere Pettersson O Niazi A Morrell JM. Metagenomic analysis of bacteria in stallion semen. Anim Reprod Sci. (2020) 221:106568. 10.1016/j.anireprosci.2020.106568 32861118

[16] Dardente H Lomet D Robert V Decourt C Beltramo M Pellicer-Rubio MT. Seasonal breeding in mammals: from basic science to applications and back. Theriogenology. (2016) 86:324–32. 10.1016/j.theriogenology.2016.04.045 27173960

[17] Fatet A Pellicer-Rubio MT Leboeuf B. Reproductive cycle of goats. Anim Reprod Sci. (2011) 124:211–9. 10.1016/j.anireprosci.2010.08.029 20888155

[18] Chemineau P Guillaume D Migaud M Thiéry JC Pellicer-Rubio MT Malpaux B. Seasonality of reproduction in mammals: intimate regulatory mechanisms and practical implications. Reprod Domest Anim. (2008) 43:40–7. 10.1111/j.1439-0531.2008.01141.x 18638103

[19] López-Sebastián A Coloma MA Toledano A Santiago-Moreno J. Hormone-free protocols for the control of reproduction and artificial insemination in goats. Reprod Dom Anim. (2014) 49:22–9. 10.1111/rda.12394 25277429

[20] Giriboni J Lacuesta L Ungerfeld R. Continuous contact with females in estrus throughout the year enhances testicular activity and improves seminal traits of male goats. Theriogenology. (2017) 87:284–9. 10.1016/j.theriogenology.2016.09.004 27707547

[21] Roca J Martínez E Sánchez-Valverde MA Ruiz S Vázquez JM. Seasonal variations of semen quality in male goats: study of sperm abnormalities. Theriogenology. (1992) 38:115–25. 10.1016/0093-691X(92)90223-E 16727123

[22] Roca J Martínez E Vázquez JM Coy P. Characteristics and seasonal variations in the semen of Murciano-Granadina goats in the Mediterranean area. Anim Reprod Sci. (1992) 29:255–62. 10.1016/0378-4320(92)90038-F

[23] Wang W Luo J Sun S Xi L Gao Q Haile AB. The effect of season on spermatozoa motility, plasma membrane and acrosome integrity in fresh and frozen-thawed semen from Xinong Saanen bucks. Reprod Domest Anim. (2015) 50:23–8. 10.1111/rda.12444 25366190

[24] Zarazaga LA Guzmán JL Domínguez C Pérez MC Prieto R. Effects of season and feeding level on reproductive activity and semen quality in Payoya buck goats. Theriogenology. (2009) 71:1316–25. 10.1016/j.theriogenology.2009.01.007 19249088

[25] Mocé E Lozano-Palazón SA Martínez-Granell MA Mocé ML Gómez EA. Effect of the refrigeration system on in vitro quality and in vivo fertility of goat buck sperm. Animals. (2020) 10:2399. 10.3390/ani10122399 PMC7765386 33333971

[26] Zarazaga LA Gatica MC Delgado-Pertíñez M Hernández H Guzmán JL Delgadillo JA. Photoperiod-treatment in Mediterranean bucks can improve the reproductive performance of the male effect depending on the extent of their seasonality. Animals. (2021) 11:400. 10.3390/ani11020400 33562447 PMC7915632

[27] BOE. Real Decreto 53/2013, de 1 de febrero, por el que se establecen las normas básicas aplicables para la protección de los animales utilizados en experimentación y otros fines científicos, incluyendo la docencia. BOE (2013). Available online at: https://www.boe.es/boe/dias/2013/02/08/pdfs/BOE-A-2013-1337.pdf (accessed November 2, 2020).

[28] Silvestre MA Salvador I Sánchez JP Gómez EA. Effect of changing female stimulus on intensive semen collection in young Murciano-Granadina male goats. J Anim Sci. (2004) 82:1641–5. 10.2527/2004.8261641x 15216989

[29] Paschoal AFL Luther AM Jäkel H Scheinpflug K Mühldorfer K Bortolozzo FP. Determination of a cooling-rate frame for antibiotic-free preservation of boar semen at 5°C. PLoS ONE. (2020) 15:e0234339. 10.1371/journal.pone.0234339 32516324 PMC7282664

[30] Peña FJ Ball BA Squires EL. A new method for evaluating stallion sperm viability and mitocondrial membrane potential in fixed semen samples. Cytometry Part B. (2018) 94B:302–11. 10.1002/cyto.b.21506 28033647

[31] Bolyen E Rideout JR Dillon MR Bokulich NA Abnet CC Al-Ghalith GA. Reproducible, interactive, scalable and extensible microbiome data science using QIIME 2. Nat Biotechnol. (2019) 37:852–7. 10.1038/s41587-019-0209-9 31341288 PMC7015180

[32] Callahan BJ McMurdie PJ Rosen MJ Han AW Johnson AJA Holmes SP. DADA2: High-resolution sample inference from Illumina amplicon data. Nat Methods. (2016) 13:581–3. 10.1038/nmeth.3869 27214047 PMC4927377

[33] Mair P Wilcox RR. Robust statistical methods in R using the WRS2 package. Behav Res Meth. (2020) 52:464–88. 10.3758/s13428-019-01246-w 31152384

[34] Arrébola FA Pérez-Marín CC Santiago-Moreno J. Limitation of seasonality in reproductive parameters of Mediterranean bucks, using photoperiod treatment. Small Rumin Res. (2010) 89:31–5. 10.1016/j.smallrumres.2009.11.016

[35] Arrébola F Abecia JA. Effects of season and artificial photoperiod on semen and seminal plasma characteristics in bucks of two goat breeds maintained in a semen collection center. Vet World. (2017) 10:521–5. 10.14202/vetworld.2017.521-525 28620256 PMC5465766

[36] González-Marín C Roy R López-Fernández C Díez B Carabaño MJ Fernández JL. Bacteria in bovine semen can increase sperm DNA fragmentation rates: A kinetic experimental approach. Anim Reprod Sci. (2011) 123:139–48. 10.1016/j.anireprosci.2010.11.014 21168290

[37] Farahani L Tharakan T Yap T Ramsay JW Jayasena CN Minhas S. The semen microbiome and its impact on sperm function and male fertility: A systematic review and meta-analysis. Andrology. (2021) 9:115–44. 10.1111/andr.12886 32794312

[38] Díaz JM Prieto A López G Díaz P López C Quintela LA. Association of *Ureaplasma diversum* with reproductive disease in cattle. N Z Vet J. (2019) 67:249–56. 10.1080/00480169.2019.1623733 31131738

[39] Machado VS Oikonomou G Bicalho ML Knauer WA Gilbert R Bicalho RC. Investigation of postpartum dairy cows' uterine microbial diversity using metagenomic pyrosequencing of the 16S rRNA gene. Vet Microbiol. (2012) 159:460–9. 10.1016/j.vetmic.2012.04.033 22595139

[40] Quereda JJ Barba M Mocé ML Gomis J Jiménez-Trigos E García-Muñoz A. Vaginal microbiota changes during estrous cycle in dairy heifers. Front Vet Sci. (2020) 7:371. 10.3389/fvets.2020.00371 32719814 PMC7350931

[41] Tiago G Carvalheira J Rocha A. Conception rate, uterine infection and embryo quality after artificial insemination and natural breeding with a stallion carrier of *Pseudomonas aeruginosa*: a case report. Acta Vet Scand. (2012) 54:20. 10.1186/1751-0147-54-20 22458304 PMC3349612

[42] Zecchinon L Fett T Desmecht D. How *Mannheimia haemolytica* defeats host defence through a kiss of death mechanism. Vet Res. (2005) 36:133–56. 10.1051/vetres:2004065 15720968

[43] Barba M Martínez-Boví R Quereda JJ Mocé ML Plaza-Dávila M Jiménez-Trigos E. Vaginal microbiota is stable throughout the estrous cycle in Arabian mares. Animals. (2020) 10:2020. 10.3390/ani10112020 PMC7692283 33153053

[44] Husso A Jalanka J Alipour MJ Huhti P Kareskoski M Pessa-Morikawa T. The composition of the perinatal intestinal microbiota in horse. Sci Rep. (2020) 10:441. 10.1038/s41598-019-57003-8 31949191 PMC6965133

[45] Eribe ERK Olsen I. *Leptotrichia* species in human infections. Anaerobe. (2008) 14:131–7. 10.1016/j.anaerobe.2008.04.004 18539056

[46] Palmer R Fleming GTA Glaeser S Semmler T Flamm A Ewers C. Marine mammals are natural hosts of *Oceanivirga salmonicida*, a bacterial pathogen of Atlantic salmon. Dis Aquat Org. (2020) 139:161–74. 10.3354/dao03478 32406871

[47] Peña-Cearra A Belanche A González-López M Lavín JL Pascual-Itoiz MA Jiménez E. Peripheral blood mononuclear cells (PBMC) microbiome is not affected by colon microbiota in healthy goats. Anim microbiome. (2021) 3:28. 10.1186/s42523-021-00091-7 33853683 PMC8048065

[48] Zhang X Wu J Zhou C Tan Z Jiao J. Spatial and temporal organization of jejunal microbiota in goats during animal development process. J Appl Microbiol. (2021) 131:68–79. 10.1111/jam.14961 33300169

[49] Guo J Li P Liu S Miao B Zeng B Jiang Y. Characterization of the rumen microbiota and volatile fatty acid profiles of weaned goat kids under shrub-grassland grazing and indoor feeding. Animals. (2020) 10:176. 10.3390/ani10020176 PMC7070841 31972989

[50] Kim KK Lee JS Stevens DA. Microbiology and epidemiology of *Halomonas* species. Future Microb. (2013) 8:12. 10.2217/fmb.13.108 24266356

[51] Althouse GC. Sanitary procedures for the production of extended semen. Reprod Domest Anim. (2008) 43:374–8. 10.1111/j.1439-0531.2008.01187.x 18638149

[52] Kuster CE Althouse GC. The impact of bacteriospermia on boar sperm storage and reproductive performance. Theriogenology. (2016) 85:21–6. 10.1016/j.theriogenology.2015.09.049 26525397

[53] Ungerfeld R Giriboni J. Freitas-de-Melo A, Lacuesta L. Homosexual behavior in male goats is more frequent during breeding season and in bucks isolated from females. Horm Behav. (2014) 65:516–20. 10.1016/j.yhbeh.2014.04.013 24792347

[54] Xia YW Cornelius AJ Donnelly CG Bicalho RC Cheong SH Sonesc JL. Metagenomic analysis of the equine placental microbiome. Clin Theriogenol. (2017) 9:452. Available online at: https://www.cabdirect.org/cabdirect/abstract/20173345242

[55] Laguardia-Nascimento M Branco KMGR Gasparini MR Giannattasio-Ferraz S Leite LR Araujo FMG. Vaginal microbiome characterization of Nellore cattle using metagenomic analysis. PLoS ONE. (2015) 10:e0143294. 10.1371/journal.pone.0143294 26599789 PMC4657983

[56] Javurek AB Spollen WG Johnson SA Bivens NJ Bromert KH Givan SA. Consumption of a high-fat diet alters the seminal fluid and gut microbiomes in male mice. Reprod Fertil Dev. (2016) 29:1602–12. 10.1071/RD16119 27569192

