# Supplementary material for: Microbiota in Goat Buck Ejaculates Differs Between Breeding and Non-breeding Seasons
Source: Front Vet Sci. 2022 May 13;9:867671. doi: 10.3389/fvets.2022.867671 (PMC9136232; doi:10.3389/fvets.2022.867671)
Supplement: Supplementary file 1 [file Image_1.pdf]

Supplementary Material

A)

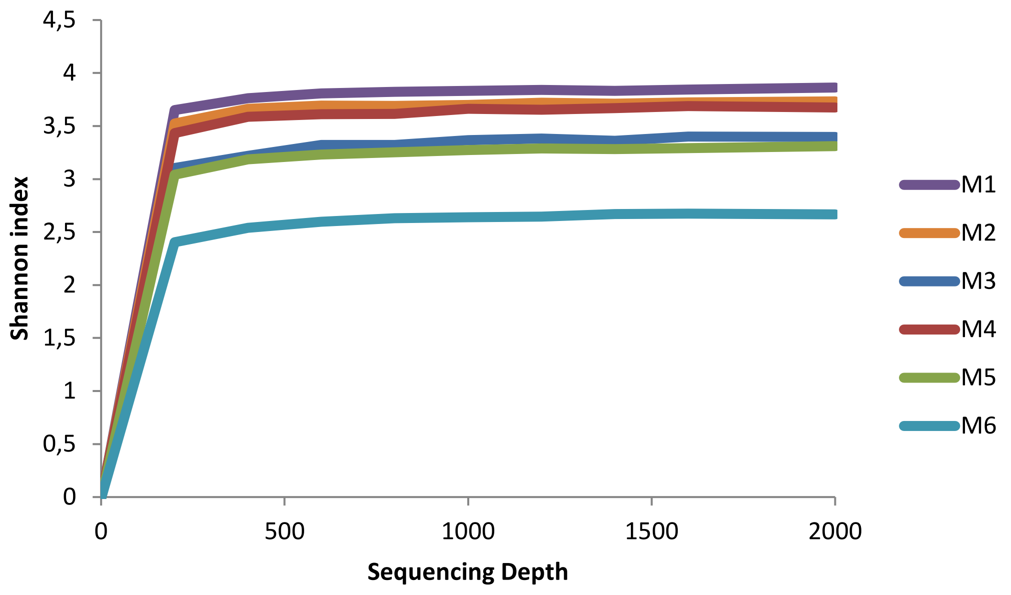

B)

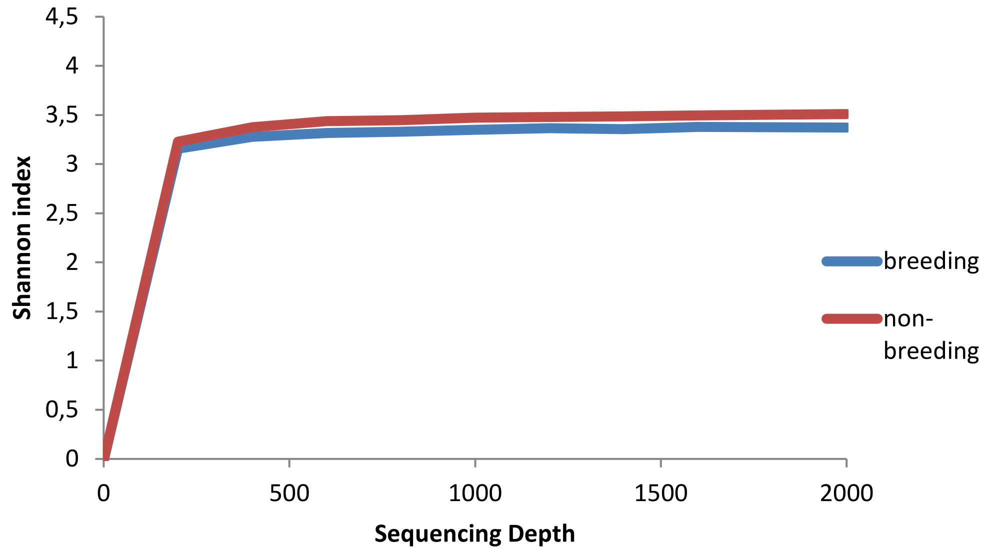

Supplementary Figure 1. Alpha diversity (Shannon index metric) for goat bucks ejaculates collected in breeding and non-breeding seasons as a function of sampling. **A)** By male; **B)** By season
